# Bactericidal Effect of Extracts and Metabolites of *Robinia pseudoacacia* L. on *Streptococcus mutans* and *Porphyromonas gingivalis* Causing Dental Plaque and Periodontal Inflammatory Diseases

**DOI:** 10.3390/molecules20046128

**Published:** 2015-04-08

**Authors:** Jayanta Kumar Patra, Eun Sil Kim, Kyounghee Oh, Hyeon-Jeong Kim, Radhika Dhakal, Yangseon Kim, Kwang-Hyun Baek

**Affiliations:** 1 School of Biotechnology, Yeungnam University, Gyeongsan, Gyeongbuk 712-749, Korea; E-Mails: jkpatra@ynu.ac.kr (J.K.P.); me_raadika@ynu.ac.kr (R.D.); 2 Wildlife Genetic Resources Center, National Institute of Biological Resources, Incheon 404-708, Korea; E-Mails: treeofieje@korea.kr (E.S.K.); kyoungheeoh@gmail.com (K.O.); 3 Ensoltek Co., Ltd., Techno 10-ro 51, Yuseong-gu, Daejeon 305-510, Korea; E-Mail: hyeonjeongkim@gmail.com

**Keywords:** fisetin, myricetin, *Porphyromonas gingivalis*, *Robinia pseudoacacia*, *Streptococcus mutans*

## Abstract

The mouth cavity hosts many types of anaerobic bacteria, including *Streptococcus* *mutans* and *Porphyromonas gingivalis*, which cause periodontal inflammatory diseases and dental caries. The present study was conducted to evaluate the antibacterial potential of extracts of *Robinia pseudoacacia* and its different fractions, as well as some of its natural compounds against oral pathogens and a nonpathogenic reference bacteria, *Escherichia coli*. The antibacterial activity of the crude extract and the solvent fractions (hexane, chloroform, ethyl acetate and butanol) of *R. pseudoacacia* were evaluated against *S.* *mutans*, *P. gingivalis* and *E. coli* *DH5α* by standard micro-assay procedure using conventional sterile polystyrene microplates. The results showed that the crude extract was more active against *P. gingivalis* (100% growth inhibition) than against *S. mutans* (73% growth inhibition) at 1.8 mg/mL. The chloroform and hexane fractions were active against *P. gingivalis*, with 91 and 97% growth inhibition, respectively, at 0.2 mg/mL. None of seven natural compounds found in *R. pseudoacacia* exerted an antibacterial effect on *P. gingivalis*; however, fisetin and myricetin at 8 µg/mL inhibited the growth of *S. mutans* by 81% and 86%, respectively. The crude extract of *R. pseudoacacia* possesses bioactive compounds that could completely control the growth of *P. gingivalis*. The antibiotic activities of the hexane and chloroform fractions suggest that the active compounds are hydrophobic in nature. The results indicate the effectiveness of the plant in clinical applications for the treatment of dental plaque and periodontal inflammatory diseases and its potential use as disinfectant for various surgical and orthodontic appliances.

## 1. Introduction

The oral cavity is a source of a variety of microorganisms that cause a series of infections and inflammation inside the cavity [1]. Films of microorganisms on the surface of teeth play important roles in the development of periodontal diseases [2]. Food debris, acid, bacteria and saliva combine in the mouth to form a sticky substance known as plaque, which adheres to the teeth [3]. *Streptococcus mutans* can colonize the surface of teeth and initiate formation of plaque by dissolving tooth structures in the presence of fermentable carbohydrates such as sucrose, fructose, and glucose using glucosyltransferase [2,4]. This occurs because the synthesis of water-insoluble glucan in the oral cavity adheres *S. mutans* and other oral microorganisms to the tooth surface, forming a barrier that prevents the diffusion of acids generated by the bacteria inside the mouth. As a result, the acids accumulate *in situ* and decalcify minerals in the enamel of the teeth, resulting in the development of pathogenic plaque [2]. The further accumulation of plaque around the gingival margin and sub gingival region of the teeth leads to a larger number of *Actinomyces* spp. and capnophilic and obligatory anaerobic bacteria, such as *Porphyromonas gingivalis* and *Prevotella intermedia* [2,5,6].

In addition to being painful, persistent dental disease is linked to diabetes, heart disease, high blood pressure and multiple sclerosis in the later part of life; therefore, there have been extensive studies conducted to control the bacteria causing dental diseases [3]. Three approaches have generally been applied to avoid diseases caused by cariogenic bacteria, inhibition of glucosyltransferase activity by specific enzyme inhibitors [2,7], inhibition of initial cell adhesion of *S. mutans* by polyclonal and monoclonal antibodies [2], and inhibition of cell growth of *S. mutans* by antibacterial agents [2]. The third approach has been extensively investigated in attempts to develop strong antibacterial agents of natural origin against these oral pathogens that could play an important role in the prevention of periodontal diseases, particularly those that affect plaque formation [8]. Many plants and their extracts have been successfully incorporated into dentifrices or mouthwashes [1,3,9,10]. Due to public concerns regarding the effects of synthetic compounds on health, public research institutes and companies are continuously engaged in studies related to testing of plant extracts and natural compounds against dominant dental pathogens such as *P. gingivalis* and *S.* *mutans* in order to develop natural remedies against these dreadful oral pathogens.

*Robinia pseudoacacia* L., which belongs to the family Fabaceae, is commonly known as black locust and is one of the most well established exotic plants in South Korea [11]. The chemical composition of *R. pseudoacacia* L. contains flavonoids including robinin (kaempferol-3-*O*-ramnozil-galactozil-7-ramnozide), acacetin-7-*O*-rutoside, apigenin, diosmetin, luteolin, secundiflorol, mucronulatol, isomucronulatol and isovestitol, which are of pharmaceutical importance [12]. These plant compounds have a number of interesting medicinal uses including antacid, antibacterial, antifungal, purgative and emenagogic effects [13,14,15].

The present study was conducted to investigate the antibacterial potential of extracts of aerial parts of *R. pseudoacacia* and its natural compounds (acacetin, amygdalin, fisetin, taxifolin, myricetin, apigenin, and rutin) against the oral pathogens, *P. gingivalis* and *S.* *mutans*, as well as a nonpathogenic *E. coli* strain. The results of this study contribute to the search for new natural antibacterial medicines and ingredients that are active against various tooth diseases particularly the compound that is responsible for its activity without affecting the normal micro flora of the oral cavity.

## 2. Results and Discussion

### 2.1. Results

The antibacterial activity of the crude extract and different fractions of *R. pseudoacacia* in terms of percentage growth inhibition against the two oral pathogenic bacteria (*P. gingivalis* and *S. mutans*) and one nonpathogenic bacteria (*E. coli* DH5α strain) are summarized in Table 1.

**Table 1:** Antibacterial activity of crude extract and fractions of *Robinia pseudoacacia* against the tested bacteria.

Plant Fractions	Concentration (mg/mL)	*P. gingivalis*	*S. mutans*	*E. coli* DH5α
		Growth Inhibition (%)		
Crude extract	0.2	88 ± 20.23 ^ab,^*^,#^	36 ± 5.79 ^x^	4 ± 0.89
	0.6	100 ± 0.09 ^a^	66 ± 8.37 ^vw^	3 ± 0.31
	1.8	100 ± 0.18 ^a^	73 ± 14.83 ^v^	1 ± 2.48
Hexane Fr.	0.008	0 ± 0.00 ^e^	11 ± 4.63 ^y^	0 ± 0.00
	0.04	60 ± 9.81 ^c^	29 ± 0.03 ^x^	0 ± 0.00
	0.2	97 ± 0.31 ^a^	55 ± 4.11 ^w^	0 ± 0.00
CHCl_3_ Fr.	0.008	10 ± 3.56 ^e^	0 ± 0.00 ^z^	4 ± 1.42
	0.04	21 ± 0.54 ^d^	2 ± 0.53 ^yz^	0 ± 0.00
	0.2	91 ± 1.80 ^ab^	31 ± 6.25 ^x^	0 ± 0.00
EtOAc Fr.	0.008	9 ± 1.05 ^e^	0 ± 3.65 ^z^	2 ± 2.76
	0.04	8 ± 3.80 ^e^	5 ± 425 ^y^	8 ± 1.39
	0.2	82 ± 3.58 ^b^	0 ± 0.00 ^z^	2 ± 1.46
BuOH Fr.	0.008	5 ± 6.72 ^e^	0 ± 0.00 ^z^	7 ± 1.36
	0.04	7 ± 3.39 ^e^	0 ± 0.00 ^z^	9 ± 1.36
	0.2	6 ± 1.79 ^e^	0 ± 0.00 ^z^	5 ± 2.94

*: Data are expressed as the mean ± SD; ^#^: Values with different superscript letters in a column are significantly different (*p* < 0.05).

At 1.8 mg/mL; the crude extract displayed a dramatic controlling effect on *P. gingivalis* (100% growth inhibition) and *S. mutans* (73% growth inhibition); but did not exert any significant effect on *E. coli* DH5α (1% growth inhibition). Even when the concentration of the crude extract was reduced by nine times (0.2 mg/mL); the extract still significantly suppressed the growth of *P. gingivalis* by 88%. The crude extract also showed high antibacterial activity against *S .mutans* at 1.8 and 0.6 mg/mL; but significantly decreased the activity at 0.2 mg/mL. The growth of non-pathogenic *E. coli* DH5α was not suppressed by any concentration of crude extract. The MIC value of the crude extract against *P. gingivalis* and *S. mutans* was found out to be 0.2 and 0.6 mg/mL respectively (Table 2).

**Table 2:** MIC value of crude extract and fractions of *Robinia pseudoacacia* against the tested bacteria.

Plant Fractions	*P. gingivalis*	*S. mutans*
Crude extract	0.2 *	0.6
Hexane Fr.	0.04	0.2
CHCl_3_ Fr.	0.2	0.2
EtOAc Fr.	0.2	>0.2 **
BuOH Fr.	>0.2	>0.2

*: Value in mg/mL; **: >—value is greater than.

The crude extract was further fractionated using four different solvents, each of which was tested from the lowest concentration of 0.2 mg/mL. At that concentration, the chloroform, ethyl acetate, and hexane fraction exerted high antibacterial activity against *P. gingivalis* with 91%, 82%, and 97%, respectively. When the concentration was diluted to 0.04 mg/mL, the chloroform and ethyl acetate fractions no longer exerted an antibacterial effect against *P. gingivalis*; however, the hexane fraction still exerted a 60% antibacterial effect (Table 1). At 0.008 mg/mL, the hexane fraction no longer exerted an antibacterial effect against *P. gingivalis*. Among the four different fractions, only the hexane fraction was active against *S. mutans*. At 0.2 mg/mL, the hexane fraction exerted 55% growth inhibition against *S. mutans*, while the crude extract exerted only 36% growth inhibition (Table 1). None of the fractions showed any significant inhibitory effect against the *E. coli* strain (Table 1). The MIC value for hexane and chloroform fraction against *P. gingivalis* and *S. mutans* was found out to be 0.04, 0.2 mg/mL and 0.2, 0.2 mg/mL respectively (Table 2).

**Figure 1:**
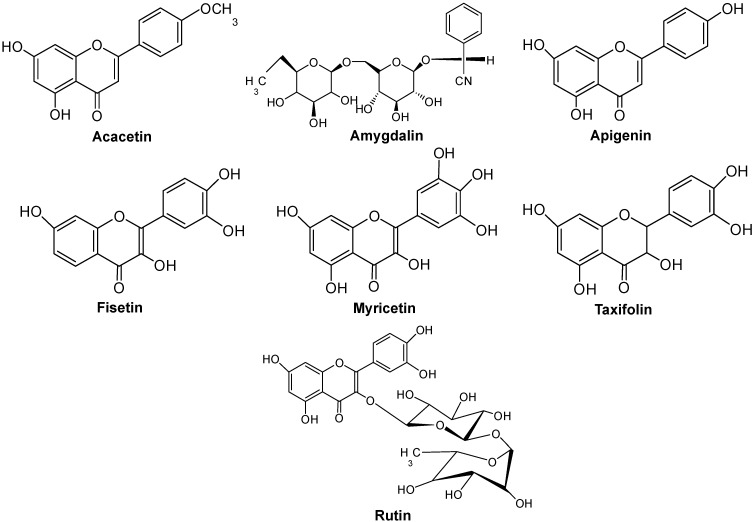
Chemical structure of the selected natural compounds in *Robinia pseudoacacia.*

**Figure 2:**
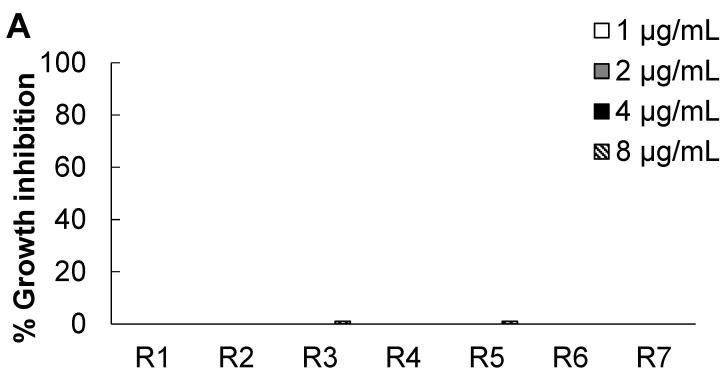

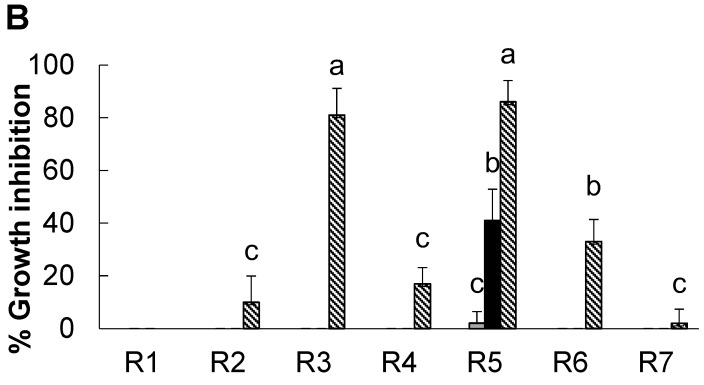

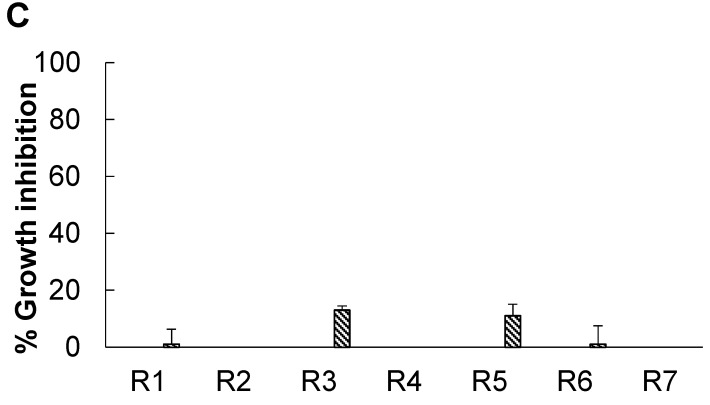
Antibacterial activity of the natural compounds in *Robinia pseudoacacia* against the tested bacteria. (**A**) *Porphyromonas gingivalis*; (**B**) *Streptococcus mutans*; (**C**) *Escherichia coli* DH5α. The natural compounds included: R1, Acacetin; R2, Amygdalin; R3, Fisetin; R4, Taxifolin; R5, Myricetin; R6, Apigenin; R7, Rutin. Different letters on the bars indicate a significant difference (*p* < 0.05).

The antibacterial activities of some natural standard compounds found in *R. pseudoacacia* L. (acacetin, amygdalin, fisetin, taxifolin, myricetin, apigenin and rutin) were tested against the pathogenic bacteria and their structures are shown in Figure 1. At 1, 2, 4 and 8 µg/mL, fisetin and myricetin exerted high antibacterial activity against *S. mutans* (Figure 2). At 8 µg/mL concentration, fisetin exerted 81% inhibition against *S. mutans*. Myricetin exerted an 86% antibacterial effect against *S. mutans* at 8 µg/mL and controlled the growth of *S. mutans* in a concentration-dependent manner. No compounds showed antibacterial activity against *P. gingivalis* (Figure 2). All compounds also did not have any significantly high inhibition activity against nonpathogenic *E. coil*.

### 2.2. Discussion

Medicinal plants have been extensively used as a source of bioactive compounds owing to their antibacterial properties against a broad range of microorganisms [16,17,18]; however, few plants have been tested against anaerobic oral pathogens [19]. *P. gingivalis* and *S. mutans* are among the two most harmful pathogenic bacteria that affect the oral cavity and thus they are widely used standard pathogenic bacteria for studying the efficacy of any plant extract or natural compound against oral pathogens [20]. Although the antimicrobial activity of *R. pseudoacacia* extracts has been reported for different pathogenic bacteria [15], the extracts have never been tested for their antibacterial effect on the oral pathogens, *P. gingivalis* and *S. mutans*. Therefore, in the present study, an attempt has been made to test the antibacterial activity of the extracts of *R. pseudoacacia* plant, which exerted the antibiotic effect on the two oral pathogenic bacteria, *P. gingivalis* and *S. mutans*, but did not exert any controlling effect on the nonpathogenic control bacterium (*E. coli* DH5α strain) (Table 1). *E. coli* is an useful bacteria that reside in the body of warm blooded organisms [21] and the lower antibacterial potential of the extracts of *R. pseudoacacia* plant is a positive sign of its potential use in the development of natural drugs. The crude extract was active against both *P. gingivalis* and *S. mutans* (Table 1) which contradicts an earlier report that *R. pseudoacacia* extracts did not have any antibacterial potential against oral pathogens [22]. The reason behind the positive antibacterial potential of the extracts of *R. pseudoacacia* might be due to the modified extraction method and use of different solvents in the extraction procedure. Apart from these, the environmental conditions also plays an important role in the varied chemical composition of any plant [23].

Next, further extractions were conducted using different solvents in an attempt to identify the active fraction(s) in which most of the antibacterial compounds were extracted from the crude extract of the *R. pseudoacacia* plant. The hexane and chloroform fractions inhibited growth of *P. gingivalis* by more than 90% (Table 1). The fractions and crude extract were more active against *P. gingivalis* than the *S. mutans* at the same concentration of 0.2 mg/mL (Table 1). This might have occurred because the thick lipopolysaccharide molecules in the Gram negative bacteria provide binding sites for the active compounds in *R. pseudoacacia*, enabling the active compounds to bind the membrane and thus resulting in cell lysis [24,25].

Phytochemical screening of various plant extracts that possessing antibacterial activity indicated the presence of different compounds such as quercetin, kaempferol, stigmasterol, campesterol, tocopherol, carotenoids, fisetin, myricetin, rutin, apigenin and many more phenolic compounds as the active compounds in them [26,27,28,29]. Further, the presence of various types of bioactive compounds in the *R. pseudoacacia* plant have also been reported [13,30,31]. Thus, based on the literature and presence of various bioactive compounds in *R. pseudoacacia*, a total of seven natural compounds possessing antibacterial potential which were earlier been reported to be present in the plant species were selected and evaluated individually for their antibacterial effect against the tested oral pathogens. Among the seven compounds tested, only fisetin and myricetin actively suppressed the growth of *S. mutans* (Figure 2). It is possible that these two compounds are extracted in hexane and chloroform as a result of which both the fractions of the plant showed antibacterial activity against *S. mutans* as compared to other fraction extracts (Table 1). Both fisetin and myricetin have been extracted from different plant sources using hexane and chloroform solvent [32,33]. Additionally, the tested compounds did not exert high antibacterial activity against *P. gingivalis* and *E. coli* at concentrations less than 8 µg/mL. Extracts of *Cotinus coggygria* that contain fisetin as one of the active compounds have been reported to possess antibacterial activity [29]. Additionally, Cai and Wu [19] reported that the compound myricetin showed antimicrobial activity against *Streptococcus mutans* and the periodontal pathogens, *P. gingivalis* and *P. intermedia*. Tsai *et al.* [26] also demonstrated that the major constituent of various herbal plants showing potent antibacterial activity against *Streptococcus sanguinis* was myricetin. Similar result on antibacterial activity of myricetin against *Pseudomonas aeruginosa* has also been reported [27]. Pimia *et al.* [34] showed that myricetin present in berries inhibited the growth of all lactic acid bacteria derived from the human gastrointestinal tract. The absence of antibacterial activity against *P. gingivalis* may be because the selected compounds in *R. pseudoacacia* were mostly flavonoids, which may not effectively control *P. gingivalis* and other compounds present in the plant might be responsible for the antibacterial activity against *P. gingivalis.*

The antibacterial effect of the plant extract justifies the potential of the plant and its ethnomedicinal use in traditional medicine [13,14]. The crude extract of *R. pseudoacacia* exerted a higher antibacterial effect on *P. gingivalis* than *S. mutans* and the different solvent fractions, indicating that the active compounds together in crude extract exerted a synergystic antibacterial activity and may inhibit the growth of *P. gingivalis* by affecting the bacterial enzymes that are responsible for the survival of the bacteria [35,36]. The result of the present study confirmed that the antibacterial potential of the extracts of the *R. pseudoacacia* plant might be due to the presence of the two compounds, fisetin and myricetin or their derivatives. These compounds act individually or synergistically against *S. mutans* by easily penetrating the bacterial cells, resulting in destruction of the cell wall and cytoplasmic membrane. The crude extract and fractions of *R. pseudoacacia* were not very effective in inhibiting the nonpathogenic bacteria (*E. coli* DH5α strain), which indicates that, if the plant extract is used as a medicine for the mouth/oral cavity or in toothpaste, it should act against oral pathogenic bacteria without affecting the normal bacterial flora.

## 3. Experimental Section

### 3.1. Plant Material

Boughs of *R. pseudoacacia* L. were collected in June 2012 from Mt. Sunuea of Gyungsang-do (Chungdo, Korea), identified by an experienced taxonomist, and processed to obtain the crude extract. The specimen of the collected *R. pseudoacacia* L. was also deposited in the Natural Products Bank, Wildlife Genetic Resources Center at NIBR. A portion of branches from the plant was selected after removing the leaves, chopped with a straw cutter (average length approximately 2.54 cm), and then dried in a drying oven for 3 days at 40 °C. Approximately 500 g of the dried plant was extracted with 80% methanol using an ultrasonic apparatus. Upon removal of the solvent under vacuum, the methanolic extract yield was 36.4 g (7.28%). The methanolic extract was then suspended in water and partitioned successively with *n*-hexane, chloroform, ethyl acetate (EtOAc) and *n*-butanol (*n*-BuOH) based on their polarity. All sample solutions were passed through a membrane filter (0.2 µm) before freeze-drying. The crude extract and fractions were subsequently stored at −20 °C freezer until further use. All chemicals used for this study were of analytical grade and purchased from Sigma-Aldrich (St. Louis, MO, USA) or Merck EMD Millipore (Billerica, MA, USA).

### 3.2. Test Pathogens

The two pathogenic bacteria, *P. gingivalis* W83 (ATCC BAA-1703^TM^) and *S.* *mutans* UA159 (ATCC 700610^TM^), and one nonpathogenic *E. coli* DH5α used in this study were obtained from the American Type Culture Collection (ATCC, Manassas, VA, USA). *P. gingivalis* was cultured anaerobically in brain-heart infusion broth supplemented with hemin and menadione, while *S. mutans* was cultured anaerobically in tryptic soy broth as previously described [37,38]. *E. coli* DH5 was incubated in Luria-Bertani (LB) broth at 37 °C.

### 3.3. Evaluation of Antibacterial Activity

The antibacterial activity of the crude and solvent fractions of the bough of *R. pseudoacacia* was evaluated against *P. gingivalis*, *S.* *mutans* and a nonpathogenic *E. coli* *DH5α* by micro-assays using conventional sterile polystyrene microplates [39]. Each well of the microplate was filled with 100 µL of sterile specific media and to it about 50 µL of inoculum and 50 µL of crude extract at the final concentrations ranging from 0.2 to 1.8 mg/mL, various solvent fractions (0.008–0.2 mg/mL) and the standard compounds (1–8 µg/mL) were added in triplicate. Media containing only 50 µL of inoculum and 50 µL of 5% dimethyl sulfoxide without the plant extract was taken as the control treatment and media containing only 50 µL of 5% dimethyl sulfoxide except the inoculum was taken as solvent control. The microplates were incubated for 24 h at 37 °C in 5% carbon dioxide atmosphere (for *P. gingivalis*, *S.* *mutans*) and normal atmospheric condition for *E. coli*. The bacterial growth was determined by the OD at 630 nm using an ELISA microplate reader (FilterMax F5 Multi-mode Microplate Reader, Molecular Devices, Sunnyvale, CA, USA). Minimum inhibitory concentration (MIC) of the extract and fractions was taken as the lower concentrations that do not show any visible growth of the organism. All the experiments were repeated three times. The percentage of bacterial growth inhibition (GI) in response to different solvent extracts was calculated from the control treatment using the following equation:

GI% = [(Control_Abs_ − Treatment_Abs_)/Control_Abs_] × 100

where Control_Abs_ is the absorbance of the control and Treatment_Abs_ is the absorbance of different extracts.

### 3.4. Statistical Analysis

All numeric data represents the means of three samples ± the standard deviation (SD). The variance of the sample data was measured by the Duncan’s test using the Statistical Analysis Software (SAS) version 9.2 (SAS Inc., Cary, NC, USA).

## 4. Conclusions

The use of plant extracts for treatment of diseases related to microorganisms has been extensively studied throughout the world. Plant extracts possess a series of bioactive compounds that have been utilized in various applications based on their biological activities. In this study, the crude extract and the two fractions (chloroform and hexane) of *R. pseudoacacia* were very active in controlling *P. gingivalis*. The individual natural compounds, fisetin and myricetin, inhibited the growth of *S. mutans*. Taken together, these results indicate that the crude extract or the fractions of *R. pseudoacacia* can be a valuable and economic resource for use in herbal toothpaste compositions or as a medicine for the treatment of dental plaque and periodontal disease. Further the plant extract and its active compounds can have potential in clinical applications for use as disinfectants for various surgical and orthodontic appliances and maintaining oral hygiene.
